# Optimization of an artificial tick feeding assay for *Dermacentor reticulatus*

**DOI:** 10.1186/s13071-017-2000-4

**Published:** 2017-02-02

**Authors:** Christoph Krull, Bettina Böhme, Peter-Henning Clausen, Ard M. Nijhof

**Affiliations:** Institute for Parasitology and Tropical Veterinary Medicine, Freie Universität Berlin, Robert-von-Ostertag-Str. 7-13, 14163 Berlin, Germany

**Keywords:** *Dermacentor reticulatus*, Artificial feeding, CO_2_, Irradiation, Glucose

## Abstract

**Background:**

The development of standardized in vitro feeding methods for ixodid ticks has been hampered by their complex feeding behaviour and the long duration of their blood meal. In this study, we aimed to optimize several parameters for the in vitro feeding of adult *Dermacentor reticulatus*.

**Methods:**

Ticks were fed on heparinized bovine blood collected at a slaughterhouse, using a modified silicone membrane feeding assay. Effects on tick feeding and fecundity of different blood meal treatments (freezing, irradiation, addition of antibiotics), ambient conditions (increased CO_2_ concentration) and phagostimulant use (addition of 2 g/l and 4 g/l glucose to the blood meal) were systematically evaluated.

**Results:**

Although fungal growth occurred more frequent in feeding units of ticks fed on defrosted blood, the attachment rate, engorgement mass and fecundity of females fed on defrosted blood did not significantly differ from that of ticks fed on fresh blood. A reduction in the fecundity of female *D. reticulatus* ticks was observed when ticks were fed with gamma-irradiated blood or untreated blood compared to blood treated with gentamycin. Both the engorgement mass and fecundity increased when ticks were fed at a 5% CO_2_ level. A non-significant increase in the engorgement mass and engorgement rate of *D. reticulatus* was observed when blood was supplemented with 4 g glucose per litre compared to 2 g/l.

**Conclusion:**

An artificial feeding method was adapted for the feeding of adult *D. reticulatus* ticks. Of all parameters tested, only the artificial feeding at 5% CO_2_ levels resulted in a significant increase (*P* < 0.05) in the engorgement mass and fecundity of female *D. reticulatus* ticks. The supplementation of blood with antibiotics resulted in a significantly higher tick fecundity in comparison to ticks fed with untreated or irradiated blood.

## Background

The development and standardisation of methods to feed ticks artificially in the laboratory, i.e. without the use of experimental animals, would contribute to the aims of 3R (Reduction, Refinement and Replacement) in animal experimentation and provide researchers with a versatile tool with many applications, for instance in drug discovery, studies on tick physiology and the kinetics of pathogen transmission [1–3]. Major constraints for the successful development of in vitro feeding methods for ticks include the complex tick feeding behaviour and the long duration of the blood meal, which may last up to several weeks [4]. Considerable progress in the in vitro tick feeding of *Ixodes ricinus* was made by the development of an artificial tick feeding system using silicone membranes [2]. Nonetheless, the attachment and engorgement rate of in vitro fed ticks is typically lower than ticks fed on experimental animals and artificially fed ticks also showed a decreased fecundity when fed for one complete generation in vitro [5]. Further optimisation and standardisation for a broad range of tick species is thus required.

*Dermacentor reticulatus* (Fabricius, 1794) is a Palaearctic tick species with a widespread but fragmented distribution in Eurasia, ranging from northern Portugal to Western Siberia [6]. Its range has rapidly expanded during the last decades, which may be explained by changes in host density, agricultural practices, and climate [7]. Adults of *D. reticulatus* show a bimodal seasonal activity and are active from late August to May, with peak activity in autumn and spring. Their preferred hosts are large mammals such as cattle, deer and dogs, and adults may occasionally bite humans [6, 8]. The short-lived immature stages are active in summer, endophilic and predominantly feed on small mammals such as rodents and insectivores [9]. *Dermacentor reticulatus* is a vector for *Babesia canis* and *Babesia caballi*, causing babesiosis in dogs and horses, respectively. It is also associated with the transmission of *Rickettsia slovaca* and *Rickettsia raoultii*, causing tick-borne lymphadenopathy (TIBOLA) in humans, and with Omsk haemorrhagic fever virus in Siberia [8, 10, 11]. Routine laboratory hosts for *D. reticulatus* include mice, guinea pigs and rabbits [12–14].

In this study, a previously described artificial feeding assay using silicone membranes [2] was adapted for the feeding of *D. reticulatus* ticks. The effects of feeding on defrosted blood, blood treated with gamma-irradiation, blood supplemented with different glucose concentrations and increased environmental CO_2_ concentrations on the engorgement and fecundity of *D. reticulatus* females were evaluated. Attempts to feed *D. reticulatus* larvae and nymphs in vitro using this system were also undertaken.

## Methods

### Ticks

Adult *D. reticulatus* were collected by flagging from the vegetation of fallow land in and near Berlin. Ticks were subsequently stored in glass containers with pierced lids in a desiccator containing saturated MgSO_4_ solution providing a relative humidity of approximately 90% [15]. The desiccator was kept at room temperature and subjected to a 15 h: 9 h light: dark cycle. A side-by-side comparison was performed for each test parameter and ticks were randomly divided over the groups.

### Measurement of hypostome length

The length of the hypostome of juvenile and adult *D. reticulatus* ticks was measured using a Leica DM2500 microscope (Leica Microsystems GmbH, Wetzlar, Germany) equipped with a DFC 425 C digital camera (Leica). The separated gnathosoma of at least 30 specimens from each life stage and sex were examined under the microscope and the distance between the tip of the hypostome and its base at the basis capituli was measured using Leica Application Suite software.

### In vivo feeding

The engorgement rates, masses and fecundity of 100 *D. reticulatus* females routinely fed on the ears of rabbits for tick colony maintenance were measured and used as a reference for the in vitro feeding data.

### Artificial feeding system

The artificial feeding system used in this study is a modified version of the in vitro feeding assay previously developed by researchers at the University of Neuchatêl [2]. Feeding units were made of borosilicate glass tubes (Neubert Glas, Geschwenda, Germany) instead of acrylic or polystyrene, and had an 28 mm inner diameter, 2 mm wall thickness and height of 65 mm. Silicone membranes were prepared by mixing 15 g Elastosil E4 silicone (Wacker Chemie, München, Germany) with 0.15 g Elastosil FL white colour paste (Wacker Chemie), 4.5 g DC 200 silicone oil (Sigma-Aldrich Chemie, München, Germany) and 2.9 g Hexane (Sigma-Aldrich) with a wooden spatula. The mixture was subsequently spread on lens cleaning paper (Tiffen, Happauge, USA) or goldbeater’s skin (Altenburger Pergament & Trommelfell GmbH, Altenburg, Germany) fixed to cling film using sticky tape and left to polymerize and dry overnight. Membranes of 80–120 μm thickness were used for adult ticks and membranes of 30–70 μm for larvae and nymphs. Membranes made with lens cleaning paper matrix were ≥ 50 μm of thickness, thinner membranes could be prepared using goldbeater’s skin. The silicone membranes were attached to the tick feeding unit using Elastosil E41 silicone glue (Wacker Chemie, München, Germany). Feeding units for adult ticks were subsequently autoclaved before use, feeding units with thinner membranes were surface sterilized in 70% ethanol for 10 min.

A square piece of glass fiber mosquito netting (Drahtwaren Driller GmbH, Freiburg, Germany), approximately 20 × 20 mm in size was glued to the silicone membrane inside the feeding units for adult ticks to provide tactile stimuli. Silicone glue was applied to two sides of the square mosquito netting only, leaving sufficient space for ticks to crawl underneath the netting.

Animal hair extract was made by incubating 50 g of animal hair with 250 ml of dichloromethane (DCM, Carl Roth GmbH, Karlsruhe, Germany) for 20 min. Hundred ml of this extract was decanted and replaced with fresh DCM. This was repeated three times, observing a 20 min incubation step after each replacement. All extracts were combined and filtered through a glass fiber filter pad (GF 6, Whatman, Maidstone, Great Britain), using a Büchner funnel and a vacuum pump. The filtrate was subsequently concentrated using a rotating evaporator to a concentration of 7 mg low volatile mass/ml and stored at -80 °C. Eighty μl of the concentrated hair extract was dispersed evenly over the silicone membrane inside the feeding units and left to dry for at least 2 h. For adult ticks, bovine and equine hair and hair extract was used, whereas immature stages were provided with mouse hair and hair extract. Conspecific tick faeces was added to each tick feeding unit as additional attachment stimuli.

### Blood

Bovine blood used for in vitro feeding was collected at a local abattoir during the exsanguination process, and directly supplemented with heparin (Ratiopharm, Ulm, Germany) (20 IU/ml) and glucose (Carl Roth GmbH, Karlsruhe, Germany). The standard glucose concentration was 2 g/l, but 4 g/l was also used for the experiment in which the effect of increased glucose concentration on in vitro tick feeding was evaluated. Blood was immediately refrigerated and stored at 4 °C for up to one week. The phagostimulant adenosine triphosphate (ATP, Carl Roth) (51 mg/ml) and the broad-spectrum antibiotic gentamycin (Roth) (5 μg/ml) were added just before use.

For experiments involving frozen blood, half of the collected blood volume was frozen at -20 °C for 24 h within four hours after collection. It was subsequently thawed at 4 °C and used for the in vitro tick feeding. The other half of the collected blood was continuously stored at 4 °C and used for feeding the control group.

For experiments in which irradiated blood was used, half of the collected blood was divided over 50 ml Falcon Tubes (Sarstedt, Nürnbrecht, Germany) and irradiated for 220 min with 266 Gy/h using a cobalt-60 source (Helmholtz- Centre Berlin for Materials and Energy, Berlin, Germany). During irradiation, the tubes were cooled using ice-packs and turned 180° after 110 min, ensuring an uniformly irradiation of the tubes and resulting in a final radiation dose of approximately 1 kGy. Bacterial growth was monitored by streaking of 100 μl irradiated or untreated blood from stock on blood agar plates in triplicate, as well as the daily streaking on blood agar plates of 20 μl blood collected from each feeding unit after it had been used for tick feeding. Blood agar plates were aerobically incubated at 37 °C for 24 h before the number of colony forming units (cfu) was determined with an upper limit of 250 cfu.

### In vitro feeding procedure

Seven female and five male *D. reticulatus* ticks were placed in each feeding unit. The feeding unit was subsequently closed by the insertion of a pierced plastic lid (PE-LD Stopfen 26 mm, Brimon Laborbedarf, Hamburg, Germany) wrapped in gauze fabric into the feeding unit, leaving approx. one cm between the membrane and the lid. The feeding unit was then hung into a glass beaker (50 ml, Simax, Czech Republic) containing the blood using a rubber ring with an inner diameter of 32 mm (Lux, Wermelskirchen, Germany).

Blood was supplemented with ATP and gentamycin as described above, and 5 ml blood per feeding unit was pipetted into a sterile beaker and preheated to 37 °C on a hot plate. The blood was changed twice daily at 12 ± 2 h intervals. During each blood change, the outside of the feeding unit and underside of the silicone membrane were rinsed with sterile 0.9% NaCl solution, pre-heated to body temperature. The number of attached ticks were counted, dead and detached engorged ticks were removed and the feeding unit was transferred to a new sterile beaker with fresh blood. Males stayed inside the feeding unit until the end of the experiment, to provide them with sufficient opportunity and time to fertilize any females present.

Feeding units were placed in a waterbath (WNE 22, Memmert, Schwabach, Germany) at 37 °C, which provided a relative humidity of approximately 78%. A lamp provided a 15 h: 9 h light: dark cycle.

For the CO_2_ and glucose experiments, feeding units were kept in an incubator (ICH 256C, Memmert), where the blood was maintained at a constant temperature of 37 °C using a heating plate (Hot Plate 062, Labotect, Göttingen, Germany). Environmental conditions were set to 20 °C, 80% RH, 5% CO_2_ (2.5% CO_2_ for the glucose experiment) and 15 h of light. Feeding units exposed to ambient CO_2_ concentrations were placed on a heating plate in the laboratory with a room temperature of about 20 °C and a 15 h: 9 h light: dark cycle. Wetted cotton balls on top of the feeding units ensured about 70% RH. Representative environmental conditions inside the feeding units were recorded by an iButton (Hygrochron, Maxim Integrated, San Jose, USA), which measured the temperature and relative humidity at 30 min intervals.

Engorged females that detached were weighed and stored individually in 2 ml Eppendorf tubes with pierced lids, which were kept in a desiccator with approximately 90% relative humidity at room temperature.

### Statistical analysis

Statistical analyses were performed using SPSS software. Engorgement rates were evaluated using Fisher’s exact test. Engorgement masses and egg batch masses were analysed using Welch *t*-test or ANOVA, depending on normal distribution and homoscedasticity of the data. The ANOVA was followed by Bonferroni *post-hoc* comparisons.

## Results

### Hypostome length

An overview of the hypostome lengths of all life stages from *D. reticulatus* is presented in Table 1.

**Table 1:** Mean length of measured hypostomes of the different life stages of *Dermacentor reticulatus*

Life stages	No. examined	Mean length of hypostome (μm)	SD (μm)
Females	32	383	27
Males	31	321	28
Nymphs	37	125	7
Larvae	34	72	5

*Abbreviation: SD* standard deviation

### In vivo feeding of *D. reticulatus*

In a small trial 64 out of 100 (64%) *D. reticulatus* females engorged with an average weight of 266.3 ± 71.4 mg (mean ± standard deviation, SD) when fed on the ears of rabbits (Table 2).

**Table 2:** Effects of different experimental parameters on the engorgement and fecundity of artificially fed *Dermacentor reticulatus* females

Experiment	Experimental group	Engorgement rate (%)	Mean engorgement mass ± SD (mg)	Females producing fertile eggs (%)	Mean egg batch mass ± SD (mg)
I	Defrosted blood	37.8 (45/119)	198.3 ± 63.7	71.1 (32/45)	95.0 ± 37.7
	Fresh blood	49.6 (59/119)	221.9 ± 63.2	71.2 (42/59)	98.3 ± 38.9
II	Untreated blood	32.0 (47/147)	222.9 ± 78.1	53.2 (25/47)^a^	74.4 ± 53.3^c^
	Irradiated blood	34.0 (50/147)	193.7 ± 64.4	56.0 (28/50)^b^	40.4 ± 29.3^cd^
	Gentamycin-treated blood	29.3 (43/147)	215.6 ± 78.3	79.1 (34/43)^ab^	94.2 ± 41.8^d^
III	Ambient CO_2_	17.0 (31/182)	220.3 ± 101.3^e^	32.3 (10/31)^f^	107 ± 60.1^g^
	5% CO_2_	25.3 (46/182)	303.9 ± 105.1^e^	82.6 (38/46)^f^	178.8 ± 86.7^g^
IV	2 g/l Glucose, 2.5% CO_2_	31.2 (24/77)	259.5 ± 71.9	87.5 (21/24)	99.8 ± 38.9
	4 g/l Glucose, 2.5% CO_2_	42.9 (33/77)	287.5 ± 68.8	87.9 (29/33)	121.3 ± 70.4
	Ticks fed on rabbits	64 (64/100)	266.3 ± 71.4	100 (64/64)	123.9 ± 54.3

*Abbreviation: SD* standard deviation

^a^Significant difference (Fisher’s exact test, *P* = 0.014)

^b^Significant difference (Fisher’s exact test (*P* = 0.027)

^c^Significant difference (ANOVA, F_(2,102)_ = 13.60, *P* < 0.001; Bonferroni *post-hoc* test, *P* = 0.005)

^d^Significant difference (ANOVA, F_(2,102)_ = 13.60, *P* < 0.001; Bonferroni *post-hoc* test, *P* < 0.001)

^e^Significant difference (Welch test, *P* = 0.001)

^f^Significant difference (Fisher’s exact test, *P* < 0.0001)

^g^Significant difference (Welch test, *P* = 0.0001)

### Artificial feeding parameters tested

#### Defrosted *vs* fresh blood

To evaluate the use of defrosted blood in comparison to blood which had not been frozen in in vitro tick feeding, adult *D. reticulatus* ticks were fed in vitro using (i) bovine blood stored at 4 °C; and (ii) blood collected from the same animal that had been frozen for 24 h, then thawed and stored at 4 °C. This experiment was performed three times, using 4, 7 and 6 feeding units per group. In the group fed with fresh blood, 59 out of 119 female *D. reticulatus* ticks engorged (49.6%) (Table 2). This was higher, but not statistically different from the group fed with defrosted blood, where 45 out of 119 ticks engorged (37.8%, Fisher’s exact test, *P* = 0.0891). The average mass of replete females was 221.9 ± 63.2 (SD) mg in the group fed with fresh blood, which was also higher, but again not significantly different from the average engorgement mass of 198.3 ± 63.7 mg) of females fed with defrosted blood (*t*-test, *t*_(102)_ = 1.882, *P* = 0.063). The average mass of egg batches laid by the engorged females was 98.3 ± 38.9 mg in the fresh blood group and 95 ± 37.7 mg in the defrosted blood group (*t*-test, *t*_(92)_ = 0.412, *P* = 0.682). The portion of engorged females that produced larval offspring was 71.2% (42/59) in the fresh blood group and 71.1% (32/45) in the frozen blood group. Fungi infestation occurred in 5.9% (1/17) of the feeding units of the fresh blood group, significantly less than the 70.6% (12/17) of affected feeding units in the frozen blood group (Fisher’s exact test, *P* = 0.0002). The average egg hatch rate was > 60% in both groups.

#### Sterilisation with antibiotics and irradiation

To evaluate possible ways to limit bacterial growth in blood used for artificial tick feeding, adult *D. reticulatus* ticks were fed in vitro using blood treated with antibiotics (gentamycin 5 μg/ml), sterilised with gamma-irradiation (~1 kGy) and untreated blood (control). This experiment was performed three times, each time with 7 feeding units per group. Colony counts of blood samples streaked on blood agar plates directly after sterilisation confirmed the efficacy of irradiation, with a mean number of 1.7 ± 3.8 (SD) cfu/ml compared to 96.1 ± 127.0 cfu/ml in untreated blood. In the group feeding on antibiotics-treated blood, 43 out of 147 females engorged (29.3%), slightly less than the number of engorged ticks from the group fed on irradiated blood (50/147; 34%) or the untreated group (47/147; 32%). The highest average engorgement mass (222.9 ± 78.1 mg) was reached in the latter group. The ANOVA test indicated group differences that were just significant at the *P* < 0.05 level (*F*_(2,137)_ = 3.13, *P* = 0.047), but subsequent analysis by the Bonferroni test could not confirm significant differences between the individual groups. However, ticks fed on blood treated with antibiotics laid significantly more eggs than both other groups, with an average egg batch mass of 94.2 ± 41.8 mg in the antibiotics group, 40.4 ± 29.3 mg in the irradiation group and 74.4 ± 53.3 mg in the untreated group (ANOVA, *F*_(2,102)_ = 13.60, *P* < 0.0001). The portion of engorged females that produced larval offspring was also significantly higher in this group (34/43; 79.1%) compared to 56% (28/50) in the irradiation group and 53.2% (25/47) in the untreated group (Fisher’s exact test, *P* = 0.019). Hatching rates were with approx. 60% similar for all groups.

#### Feeding with 5% CO_2_

To evaluate the effect of CO_2_ on tick feeding, groups of adult *D. reticulatus* ticks were simultaneously fed in vitro under atmospheric conditions (0.04% CO_2_) and in an incubator with 5% CO_2_. The experiment was performed four times, once using 8 feeding units and three times using 6 feeding units per group. In total, 364 female ticks were fed of which 77 engorged (21.1%). A larger number of ticks engorged under 5% CO_2_ conditions (46/182; 25.3%) compared to atmospheric conditions (31/182; 17%) (Fisher’s exact test, *P* = 0.0719). The average mass of engorged females, as well as the egg batch were also significantly higher in the CO_2_ group: 303.9 ± 105.1 *vs* 220.3 ± 101.3 mg (*t*-test, *t*_(75)_ = 3.474, *P* = 0.001) and 178.8 ± 86.7 *vs* 107 ± 60.1 mg (Welch-test, *P* = 0.0001). The proportion of engorged females that produced larval offspring was 32.3% (10/31) in the laboratory group and 82.6% (38/46) in the incubator group.

#### Increased glucose levels

The effect of glucose addition to the bloodmeal was evaluated by feeding blood containing 2 g (control group) and 4 g (test group) glucose per litre. This experiment was performed twice, using 5 and 6 feeding units respectively under a 2.5% CO_2_ concentration. In the test group fed with the highest amount of glucose, 33 out of 77 ticks engorged (42.9%), more than in the control group (24/77; 31.2%; Fisher’s exact test, *P* = 0.1816,). The average mass of engorged females was 287.5 ± 68.8 mg in the test group and 259.5 ± 71.9 mg in the control group (*t*-test, *t*_(55)_ = 1.485, *P* = 0.143). In the control group, 21 out of 24 females (87.5%) laid batches of fertile eggs, with an average mass of 99.8 ± 38.9 mg. This was not statistically different from the number of females which produced larval offspring in the test group (29/33, 87.9%) or the mass of egg batches from this group (121.3 ± 70.4 mg; Welch-test, *P* = 0.213). The hatching rate could unfortunately not be determined for each group separately as the ink used for labelling some of the tubes had faded during their storage at high humidity in the desiccator, but the collective hatching rate for both groups was > 80%.

## Discussion

A simple method to feed ixodid ticks artificially would provide researchers with a versatile tool to study tick biology and could reduce the number of experimental animals used in tick research, but its development is hampered by the complex feeding behaviour of hard ticks and the long duration of their blood meal. Several methods to feed ticks in vitro were developed in the past century; including methods by scientists of the University of Neuchâtel using a silicone membrane which allowed for the completion of the life cycle of *Amblyomma hebraeum* ticks and also formed the foundation for in vitro acaricide screening assays [2, 5]. In this study, we used a modified version of the latter system for the optimization of several parameters in the artificial feeding of *D. reticulatus*. Modifications included the use of glass feeding units and beakers for containment of the blood meal, as these can be autoclaved and are more sustainable than polystyrene or acrylic glass. Blood was anticoagulated using heparin rather than manual defibrination, to provide ticks with a blood meal that most closely resembles its natural composition. Results from previous studies suggested that heparin is the preferred anticoagulant for the artificial feeding of arthropods, resulting in higher engorgement masses and fecundity [16–19]; we found 20 IU/ml to be the minimum concentration that still prevented clotting (results not shown). Furthermore, the dead volume inside the tick feeding unit was restricted by the insertion of a pierced plastic lid wrapped in gauze fabric, which forced the ticks close to the silicone membrane [20].

The thickness of the silicone membrane was adjusted to the length of the tick’s mouthparts, which were measured for all life stages of *D. reticulatus* (Table 1). This is of particular relevance for the larval ticks, which have the shortest mouthparts of all life stages. The hypostome length of *D. reticulatus* larvae (72.4 μm) is comparable to that of *Rhipicephalus microplus* larvae as measured by scanning electron microscopy (78–88 μm) [21]. A short hypostome length complicates the development of an appropriate silicone membrane which is both thin enough for the larvae to pierce through, but at the same time thick enough to prevent leakage of blood into the tick feeding unit. Thinner silicone membranes could be made when goldbeater’s skin instead of lens cleaning paper was used as a matrix. The used lens cleaning paper was approx. 50 μm thick and the goldbeater’s skin used measured only 20–30 μm. Goldbeater’s skin, also referred to as baudruche membrane, is prepared from the serosa of the intestine from cattle or sheep and has previously been used as a membrane-matrix for the in vitro feeding of *Amblyomma variegatum*, *R. microplus* and *R. appendiculatus* [19, 22, 23]. However, despite extensive experimentation with different artificial membranes, including thin (~30–50 μm) silicone membranes based on a matrix of goldbeater’s skin, different attachment stimuli including mice and rabbit hair extracts, and different blood sources (cattle and rabbit) we failed to feed *D. reticulatus* larvae or nymphs in vitro in significant numbers. The feeding rates on silicone membranes were lower than 1%. Slightly higher engorgement rates of 4–7% were obtained when we used an artificial feeding system in which the blood meal was placed in a Rutledge-type feeder above the ticks and animal skins were used instead of synthetic membranes (after [24]; results not shown). A combination of both techniques, i.e. the use of animal skin instead of silicone membranes may be of interest for further follow-up studies aiming to complete the life-cycle of *D. reticulatus* in vitro.

Bovine blood used for tick feeding was collected during the exsanguination of cattle at a slaughterhouse. The use of frozen blood would limit the workload associated with artificial tick feeding, as it would avoid the necessity of regular blood collections. When defrosted blood was fed to *D. reticulatus* females, similar engorgement and fecundity rates were obtained compared to the feeding of fresh blood, although there was a tendency for higher engorgement rates when fresh blood was used (*P* = 0.0891). In addition, a larger number of feeding units exposed to defrosted blood were contaminated with fungal infections. This may be prevented by the addition of fungicides to the blood meal, but this could in turn have negative consequences for tick gut microbiota. Other alternatives for the use of fresh blood, such as portioning and freezing smaller amounts of blood that can be thawed immediately before each blood change or other preservation methods such as oven-drying [25], separation of the blood components combined with the use of preservative solutions [26] or cryopreservation remain to be investigated.

Although care was taken to collect blood during exsanguination as aseptic as possible, it was not sterile as demonstrated by the growth of colonies on blood agar plates streaked with freshly collected blood. Since the in vitro tick feeding is normally not performed under sterile conditions and the required blood temperature of approx. 37 °C [18] further facilitates bacterial growth, methods to limit the deterioration of the blood meal, such as the addition of broad-spectrum antibiotics or irradiation, are required. We evaluated both the irradiation of blood as well as the addition of gentamycin, a broad-spectrum antibiotic previously used in the artificial feeding of other tick species [2, 5, 20], on *D. reticulatus* engorgement and fecundity. Although the number of cfu/ml in blood directly after irradiation was lower than in untreated blood, bacterial growth in irradiated blood was later found to be comparable to that in untreated blood without antibiotics (results not shown). The use of both irradiated blood and blood without antibiotics for tick feeding resulted in a significant reduction in the fecundity of *D. reticulatus* females compared to females fed with gentamycin-treated blood (Table 2). Although the addition of antibiotics to the bloodmeal seems to be advantageous at first sight, it is also likely to affect the tick microbiota, which in turn may have both short- and long-term consequences for tick fitness, survival and vector competence that need to be studied in more detail [27, 28]. Alternatively, a reduction in bacterial load might also be feasible by the sterile collection of blood and strict hygienic measurements such as surface sterilisation of ticks prior to feeding and handling of the tick feeding units under aseptic conditions.

A common problem in the in vitro feeding of hard ticks is the low attachment rate compared to ticks fed on experimental animals. In our experience, the average feeding rates of *D. reticulatus* ticks on animals is 72 ± 15% for adults, and high feeding rates can also be obtained for larvae and nymphs when they are fed soon after hatching and molting. The average engorgement rate of all experiments was 31.6% (378/1197). Low in vitro- compared to in vivo feeding rates may be due to differences in the host origin of the blood meal, or the quality of blood and/or attachment and feeding stimuli. In order to improve the attachment rate, the effect of an increased CO_2_ concentration in the atmosphere on in vitro tick feeding was evaluated. Carbon dioxide was previously shown to stimulate questing behaviour in *Dermacentor variabilis* [29] and to stimulate in vitro tick feeding of all life stages of *Amblyomma variegatum* [18], a tick species which employs a hunting strategy and actively approaches its host. The location of vertebrate hosts is largely based on olfactory cues, including CO_2_ [30]. Our study showed that the in vitro feeding of *D. reticulatus* adults also improved when ticks were fed at a 5% CO_2_ level, suggesting that ticks that employ an ambush strategy, such as *D. reticulatus* adults, may also benefit from increased CO_2_ levels during in vitro feeding. In this study, a concentration of 5% CO_2_ was used, as concentrations between 5–10% were previously shown to be result in higher feeding rates and repletion masses in the in vitro feeding of all life stages of *A. variegatum* [18]. Further studies could focus on optimizing this parameter to determine the lowest CO_2_ concentration at which the beneficial effects are highest.

The storage of blood results in a depletion of glucose, which causes a destabilisation of erythrocyte cell membrane and hemolysis [31]. In addition, *Rhipicephalus sanguineus* was shown to have cheliceral sensilla that are very sensitive to glucose [32] and a saline solution containing glucose in combination with ATP or glutathione (GSH) was shown to induce a feeding response in the soft tick *Ornithodoros tholozani* [33]. We therefore hypothesized that an increase in the glucose concentration of the blood supplied to the ticks could have a beneficial effect on the feeding rate and repletion mass of *D. reticulatus* ticks. Indeed, the results of the present study show a tendency towards a positive effect of supplementation of the blood meal with additional glucose on the feeding success of *D. reticulatus*, but this effect was not statistically significant. Additional experiments with different glucose concentrations might give more decisive proof of the effects of increased glucose concentrations on in vitro tick feeding. Another approach would be the use of 2,3-diphosphoglycerate, which was shown to increase engorgement of the hematophagous bug *Rhodnius prolixus* [34], but its high costs prevented us from evaluating the use of this potential phagostimulant.

## Conclusions

We modified a previously developed artificial feeding system for *D. reticulatus* and found that the in vitro feeding under a 5% CO_2_ concentration significantly improved the feeding rate and increased the engorgement masses of *D. reticulatus* females. The addition of a broad-spectrum antibiotic to the blood meal resulted in a significantly higher fecundity of the artificially fed females compared to ticks fed with untreated or irradiated blood. However, the effect of feeding antibiotics-treated blood on the tick’s microbiome and the long-term fitness and fecundity of ticks remains to be determined. The use of fresh instead of defrosted blood and blood supplemented with 4 g glucose per litre instead of 2 g/l tended to give better feeding rates and engorgement masses, but these results were not significant. We were not able to feed juvenile *D. reticulatus* ticks in significant numbers using this system and there is still room for further improvement of the technique, for instance by testing additional attachment and feeding stimuli or alternative membranes such as animal skin. With two blood changes per day, the in vitro feeding of ticks is laborious and automation of the process would also be desirable.
